# Functional Roles of DYRK2 as a Tumor Regulator

**DOI:** 10.3390/cimb45100538

**Published:** 2023-10-23

**Authors:** Yuta Mochimaru, Kiyotsugu Yoshida

**Affiliations:** Department of Biochemistry, The Jikei University School of Medicine, Tokyo 105-8461, Japan; yuta.mochimaru@jikei.ac.jp

**Keywords:** DYRK2, solid tumor, non-solid tumor

## Abstract

The dual-specificity tyrosine phosphorylation-regulated kinase 2 (DYRK2) regulates the induction of apoptosis and DNA repair, metastasis inhibition, cell cycle G1/S transition, protein scaffold stability for E3 ligase complexes, and embryogenesis. Owing to these functions, DYRK2 is thought to regulate tumorigenesis, and its function in cancer has been investigated. Notably, DYRK2 has been reported to function as a tumor suppressor; however, it has also been reported to act as an oncogene in some cancers. This discrepancy makes it difficult to elucidate the conserved functions of DYRK2 in cancer. Here, we reviewed the functions of DYRK2 in various cancers. Patient tissue samples were evaluated for each cancer type. Although some studies have used cell lines and/or xenografts to elucidate the mechanism of DYRK2 function, these studies are not sufficient to understand the role of DYRK2 in cancers. In particular, studies using genetically modified mice would help us to understand the reported functional duality of DYRK2 in cancer.

## 1. Introduction

Cancer is one of the most feared diseases worldwide. Currently, several cancer treatments are available, including surgery, laser treatment, chemotherapy, and gene therapy. However, they have been unsuccessful in achieving a complete cure in most patients. It is well known that gene mutations trigger tumorigenesis, and they have been characterized in all cancers. There are two types of cancer-regulating genes: tumor suppressors and oncogenes. Tumor suppressors inhibit cancer growth, while oncogenes promote it. Both of these genes can potentially be targets for cancer drugs; therefore, they are key genes in cancer treatment.

Dual-specificity tyrosine phosphorylation-regulated kinase 2 (DYRK2) was reported as one of the seven DYRK1 isoforms by Becker et al. in 1998 [1]. DYRK2 is located on chromosome 12q15, and six splicing variants have been identified [2]. DYRK2 is involved in cell survival, development, differentiation, gene transcription, proteasomal regulation, and microtubule formation [2,3,4]. These functions might contribute to cancer by affecting the cell cycle, apoptosis, epithelial–mesenchymal transition (EMT), metastasis, cancer stem cell (CSC) formation, and cell survival [2,5,6,7,8]. Several substrates phosphorylated by DYRK2 have been reported, including cellular myelocytomatosis oncogene product (c-Myc), c-Jun, SNAIL, TP53, and telomerase reverse transcriptase (TERT) [2,5]. The first paper on DYRK2 in cancer was published in 2003, and it was reported that DYRK2 mRNA amplification and overexpression were detected using oligonucleotide microarrays in lung adenocarcinomas [9]. Since then, the number of DYRK2 studies in cancer has increased [2,10,11]. Furthermore, DYRK2 may regulate both solid and non-solid tumors [12,13,14]. However, the role of DYRK2 in cancer remains controversial as there are two opinions regarding DYRK2 function. Some studies have reported that DYRK2 is a tumor suppressor in many cancer types, whereas others have reported that it is an oncogene in lung, esophageal, ovarian, prostate, and breast cancers [2,10]. The reported duality in the functions of DYRK2 in various cancers has complicated our understanding of DYRK2 function in tumors. To target DYRK2 in cancer treatment, DYRK2 function must be clarified. Here, we review the functions of DYRK2 in various cancers and elucidate the current understanding.

## 2. DYRK2 in Various Cancers

### 2.1. Colorectal Cancer

DYRK2 mRNA expression in primary or metastatic colorectal cancer is lower than that in normal colon tissue or non-metastatic colorectal cancer [15]. In addition, according to Oncomine database analysis, the incidence of high-grade colorectal cancer and metastatic colorectal cancer in the liver is lower than that of low-grade and non-metastatic colorectal cancers [15].

Microarray data from patients with colorectal cancer showed that 65.06% of tumor samples had low DYRK2 mRNA expression, whereas only 34.94% of the adjacent paracancerous tissue samples had low DYRK2 mRNA levels [16]. Additionally, DYRK2 protein expression levels in colorectal cancer tissues are lower than those in adjacent non-cancerous tissues [15,17,18]. Furthermore, DYRK2 mRNA levels were lower in metastatic and non-metastatic colorectal cancer tissues and three cell lines (HT29, SW1116, and SW480) than in non-colorectal cancer tissues and a normal human colon epithelial cell line (NCM460) [19].

The overall two-, three-, and five-year accumulative survival rates of patients with high DYRK2 expression were 81.4%, 77.4%, and 75.4%, respectively, whereas those of patients with low DYRK2 expression were 77.4%, 65.6%, and 44.3%, respectively [15]. DYRK2 expression is significantly correlated with both disease-free and overall survival, and patients with high DYRK2 expression have better survival than those with low DYRK2 expression [16,20]. Notably, DYRK2 expression was related to the clinicopathological features of age and tumor sites but not to sex, clinical stage, tumor-node-metastasis (TNM) classification, or pathological differentiation [17]. However, another study reported that DYRK2 expression and clinicopathological features were strongly associated with tumor site, stage, nodal classification, metastasis, and mortality, but not with sex, age, or tumor differentiation [15]. Therefore, the relationship between DYRK2 expression and the clinicopathological features remains controversial and requires further investigation.

Interestingly, a correlation was observed between metastasis and DYRK2 expression. Twist, an EMT transcription factor, is inversely correlated with DYRK2 expression in colorectal cancer samples [16], and miR-622, a tumor-related gene, is also reported to be inversely correlated with DYRK2 [19]. DYRK2 expression and a combination of DYRK2 and Twist expression are predictors of liver metastasis and clinical prognosis, respectively, in patients with colorectal cancer [16,20].

DYRK2 overexpression in HCT116 and RKO cells induces p53-Ser46 phosphorylation, apoptosis, cleaved poly (ADP-ribose) polymerase (PARP), and caspase 3 [21]. In contrast, c-Myc and Cyclin D1 and D2 expression levels are suppressed by DYRK2 overexpression, which inhibits cell growth and colony formation [21]. Another study reported that DYRK2 overexpression inhibits cell proliferation and colony formation by suppressing c-Myc expression, cell migration, and invasion in a kinase activity-dependent manner [20]. Cell migration and invasion are essential for EMT, and EMT is caused by changes in the expression of E-cadherin, N-cadherin, and Snail. E-cadherin expression increases, whereas Snail expression decreases in DYRK2-overexpressed cells [20].

DYRK2 is associated with the anti-cancer effect of 5-fluorouracil (5-FU). DYRK2 overexpression in 5-FU-resistant (5-FUR) LoVo cells inhibited cell proliferation, migration, and invasion; induced apoptosis; impaired viability; and enhanced sensitivity to 5-FU treatment over 24 h [16]. Moreover, 5-FUR LoVo cells showed a lower expression of DYRK2 and Vimentin, a mesenchymal marker, than normal LoVo cells and exhibited a spindle-shaped morphology [16]. Twist protein levels are reduced in DYRK2-overexpressed 5-FUR LoVo cells, promoting the ubiquitination of Twist [16].

Regarding miR-622, when DYRK2 expression was suppressed, miR-622 expression increased in colorectal tissue samples and cell lines [19]. Colorectal cancer cells with downregulated miR-622 display inhibited cell migration and invasion [19].

According to a ChIP assay, one DYRK2 promoter region is located in the-150/+1 region, which contains a large proportion of cytosine and guanine CpG islands [17]. 5-Azacytidine (Aza), a DNA methyltransferase inhibitor, upregulates DYRK2 mRNA expression in some cell lines, and DYRK2 protein upregulation has been detected in HCT116 cells [17]. In contrast, DYRK2 knockdown promoted HCT116 cell proliferation, which increased upon Aza treatment [17]. In HCT116 cells, Aza treatment reduces DNA methyltransferase 1 (DNMT1) protein expression, and DNMT1 knockdown induces DYRK2 upregulation [17]. In addition, the DYRK2 promoter region was more highly methylated in human colorectal cancer tissues than in adjacent non-colorectal cancer tissues [17].

Studies have also been conducted in mouse xenograft models. In a xenograft study, tumor growth rate, volume, and weight were inhibited by DYRK2 overexpression [16]. DYRK2 overexpression using an adenovirus vector also suppressed tumor size and weight, significantly reduced the proportion of Ki-67, a cell proliferation marker, and increased cleaved caspase 3, an apoptosis induction marker [21].

**Figure 1:**
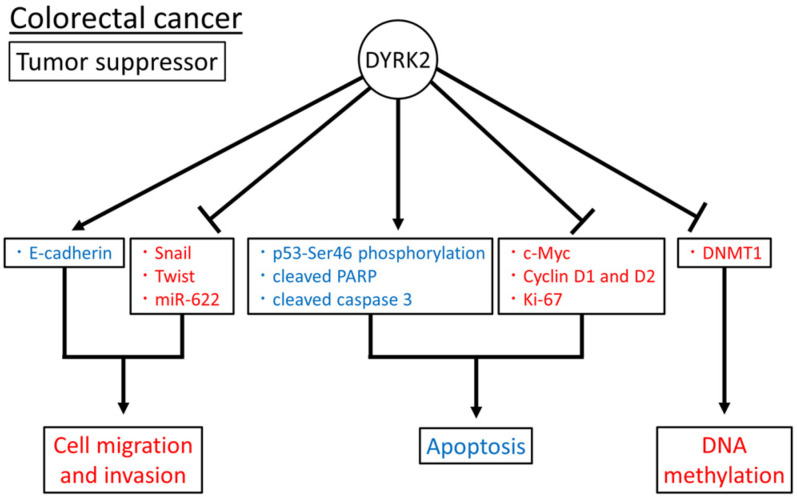
Reported DYRK2 substrates and functions in colorectal cancer. Induced and inhibited substrates or functions of DYRK2 are in blue and red, respectively. The arrow means promotion and the bar means inhibition respectively.

Together, DYRK2 as a tumor suppressor may induce apoptosis by inducing p53-Ser46 phosphorylation; cleaved PARP and caspase 3; the suppression of c-Myc, Cyclin D1 and D2, and Ki-67 expression; the inhibition of cell migration and invasion by regulating E-cadherin, Snail, Twist, and miR-622 expression; and the regulation of DNA methylation by DNMT1 expression (Figure 1). The role of DYRK2 in colorectal cancer has been investigated in tissue samples, cell lines, and xenograft mouse models. However, to date, no such studies have been conducted using genetically modified mice.

### 2.2. Liver Cancer

Patients with hepatocellular carcinoma (HCC) and low DYRK2 expression have significantly shorter survival times than those with high DYRK2 expression [22]. Another study reported that DYRK2 is an important predictor of overall survival in patients with HCC and that the inhibition of DYRK2 expression is related to poor prognosis [23]. Therefore, DYRK2 expression may serve as a predictor of liver cancer.

There is an inverse relationship between DYRK2 and c-Myc in HCC [24]. Patients with low DYRK2 and high c-Myc expression have shorter survival times, and vice versa [24]. In addition, low DYRK2 and high c-Myc expression in patients with HCC are associated with the clinicopathological features of cirrhosis, older age, and poorly differentiated carcinoma [24]. DYRK2 expression is decreased in liver cancer cells and is strongly related to the pathological grade of patients with liver cancer [23]. Moreover, DYRK2 depletion promotes liver cancer cell proliferation and increases oxaliplatin resistance [23].

DYRK2 knockdown in Huh1 and PLC/PRF5 cells resulted in cyclin D1, cyclin D2, and c-Myc accumulation, thereby promoting cell proliferation [22]. In contrast, DYRK2 overexpression in Huh1 and PLC/PRF5 cell lines using an adenoviral vector suppressed cyclin D1 and D2 and c-Myc expression, tumor growth, and colony formation [22]. DYRK2 overexpression was also investigated in another study, in which its overexpression in Huh1 and PLC/PRF5 cell lines using an adenoviral vector decreased sphere formation and ATP production by glycoses. Therefore, DYRK2 is involved in stemness acquisition and metabolic reprogramming of these cells [24]. Additionally, DYRK2 overexpression suppressed c-Myc and HRAS protein expression in a kinase activity-dependent manner [24]. The cell cycle of these DYRK2 knockdown cell lines differed from that of control cells; specifically, the G1 population was reduced and the S phase increased [22]. Furthermore, DYRK2 overexpression increased the G1 phase population, decreased the S and G2 phase populations, and induced apoptosis through p53-Ser46 phosphorylation in a kinase activity-dependent manner [22].

A mouse xenograft model study revealed that the size and weight of the inoculated DYRK2 knockdown Huh1 and PLC/PRF5 cell lines were significantly larger than those of the control cells four weeks after inoculation [22]. In contrast, xenograft tumors injected with DYRK2 adenoviral vectors had significantly lower tumor sizes and weights than control cells, and these xenograft tumors showed decreased ki-67 expression and increased cleaved caspase 3 expression, and terminal deoxynucleotidyl transferase dUTP nick end labeling [22].

We conducted a liver cancer study using a genetically modified mouse model. DYRK2 expression was decreased through hydrodynamic tail vein injection (HTVi) with a Sleeping Beauty transposon and three oncogenes, myristoylated Akt, c-Myc, and Hras^G12V^ (SB mouse model) and through the intraperitoneal injection of diethylnitrosamine with a choline-deficient l-amino acid-defined high-fat diet [24]. The SB mouse model developed HCC two weeks after injection; however, this dramatic hepatocarcinogenesis was suppressed by DYRK2 expression [24].

**Figure 2:**
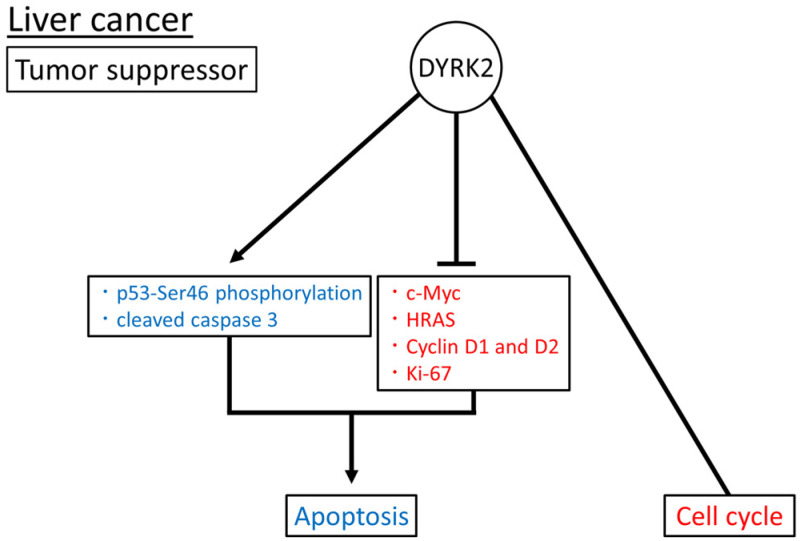
Reported DYRK2 substrates and functions in liver cancer. Induced and inhibited substrates or functions of DYRK2 are in blue and red, respectively. The arrow means promotion and the bar means inhibition respectively.

Overall, DYRK2, as a tumor suppressor, may induce apoptosis by inducing cleaved caspase 3 and p53-Ser46 phosphorylation; suppressing c-Myc, HRAS, Cyclin D1 and D2, and Ki-67 expression; and regulating the cell cycle (Figure 2). The role of DYRK2 in liver cancer has been investigated in patient tissue samples, cell lines, xenograft mouse models, and genetically modified mouse models.

### 2.3. Gastric Cancer

Low DYRK2 expression, older age, TNM stages (especially later TNM, T, or N stages), and high preoperative carcinoembryonic antigen levels correlate with poor five-year survival rates in patients with gastric cancer [25]. Higher DYRK2 expression, lower TNM stage, and younger age are associated with a better prognosis [25].

DYRK2 knockout or knockdown gastric cancer cell lines, MKN1 and AGS, showed increased proliferation, whereas a DYRK2-overexpressed gastric cancer cell line, HGC-27, showed decreased proliferation [25]. DYRK2 inhibited EMT because DYRK2 knockdown cells showed increased S100A4 and EMA levels, and DYRK2 overexpression cells showed increased E-cadherin levels and decreased S100A4 and N-cadherin levels [25].

MKN1 cells were subcutaneously injected into nude mice, and DYRK2 overexpression tumors were significantly smaller than control tumors [25]. The DYRK2 overexpression tumors were lymphocytes, and the control tumors were mostly cancer cells [25]. In contrast, DYRK2 knockout cells show the decreased expression of Beclin1 and LC3 autophagy markers, while DYRK2 knockdown cells show decreased LC3 expression [25]. DYRK2 knockdown in MKN1 cells significantly decreased LC3 expression, whereas DYRK2 overexpression significantly increased LC3 expression [25]. Thus, DYRK2 induces autophagy in gastric cancer [25].

**Figure 3:**
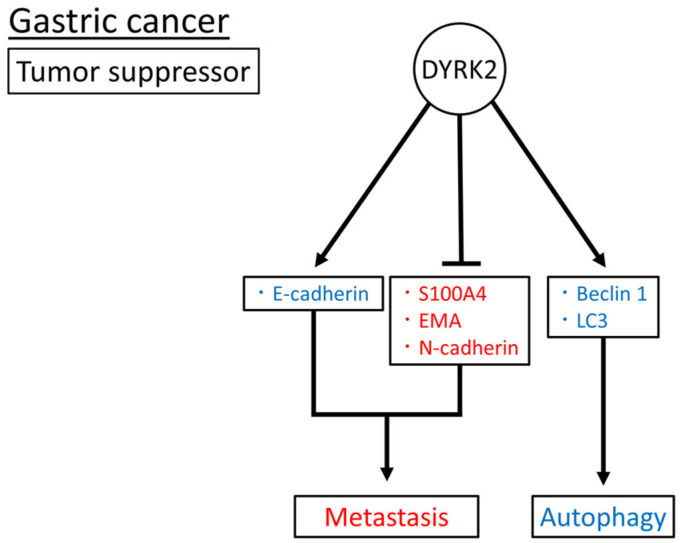
Reported DYRK2 substrates and functions in gastric cancer. Induced and inhibited substrates or functions of DYRK2 are in blue and red, respectively. The arrow means promotion and the bar means inhibition respectively.

Taken together, DYRK2, as a tumor suppressor, may inhibit metastasis by inducing E-cadherin expression, suppressing S100A4, EMA, and N-cadherin expression, and promoting autophagy by increasing Beclin1 and LC3 expression (Figure 3). The role of DYRK2 in gastric cancer has been investigated in patient tissue samples, cell lines, and xenograft mouse models. However, to date, no such studies have been conducted using genetically modified mice.

### 2.4. Non-Solid Tumors

DYRK2 levels are low in chronic myeloid leukemia (CML) cell lines (K562, KU-812, and KBM5), whereas DYRK2 expression is high in hematopoietic stem cells (lineage-negative Sca-1^+^ c-Kit^+^ (LSK) cells) from Krüppel-like factor 4 (KLF4) knockout mice [12]. KLF4 regulates stem cell self-renewal and reprogramming [26,27,28,29,30,31,32]. KLF4 inhibits *DYRK2* gene expression by binding to the endogenous *DYRK2* promoter and plays a role in maintaining c-Myc expression in CML stem/progenitor cells [12]. In addition, DYRK2 upregulation induces p53 phosphorylation at Ser46 and reduces c-Myc protein levels in mouse LSK [12]. Therefore, DYRK2 may play a role in the self-renewal and survival of stem/progenitor cells.

DYRK2 mRNA and protein levels are reduced in tissue samples from patients with non-Hodgkin’s lymphoma (NHL), and low DYRK2 expression is associated with poor prognosis in patients with NHL [14].

**Figure 4:**
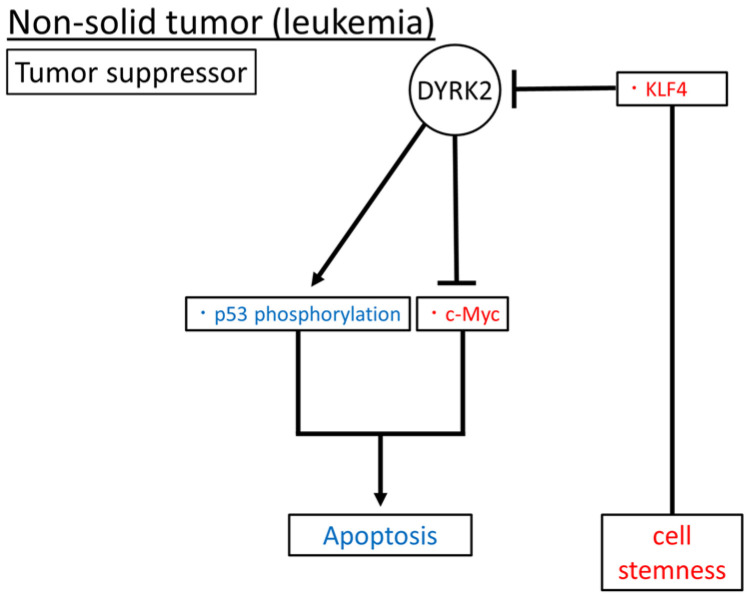
Reported DYRK2 substrates and functions in non-solid tumors (leukemia). Induced and inhibited substrates or functions of DYRK2 are in blue and red, respectively. The arrow means promotion and the bar means inhibition respectively.

Taken together, DYRK2, as a tumor suppressor, may induce apoptosis via the p53 phosphorylation and suppression of c-Myc expression and regulate cell stemness through an inhibitory interaction with KLF4 in leukemia (Figure 4). In addition, DYRK2 expression may correlate with the prognosis of patients with lymphoma. Notably, the role of DYRK2 in leukemia has been investigated in cell lines and primary cultures, while its role in lymphoma has been studied only in patient samples.

### 2.5. Cancer Stem Cells (CSCs)

CD44 and CD24 are cell surface markers, and CD44^+^/CD24^−^ is characteristic of stem cells. DYRK2 expression was inversely correlated with CD44^+^/CD24^−^ subpopulations and mammosphere formation in breast cancer specimens and cell lines [33]. In cell lines, KLF4 expression inversely correlated with DYRK2 expression and induced tumor formation in a xenograft mouse model [33]. Therefore, DYRK2 regulates CSC population via KLF4 expression [33].

Taken together, DYRK2 as a tumor suppressor may regulate cell stemness and tumor formation through an inhibitory interaction with KLF4. Notably, the role of DYRK2 has been investigated in cell lines and a xenograft mouse model. However, to date, no such studies have been conducted on patient samples or genetically modified mice.

### 2.6. Breast Cancer

DYRK2 has been reported to function as both a tumor suppressor and an oncogene in breast cancer.

Breast cancer tissue samples showed markedly lower DYRK2 expression than the normal tissue samples [18]. DYRK2 expression levels in patient tissue samples have an inverse relationship with c-Jun, c-Myc, and cyclin E accumulation, and more than 50% of patient tissue samples with high DYRK2 expression were positive for phospho-c-Jun at Ser243 and phospho-c-Myc at Ser62 [18].

DYRK2 and KLF4 expression are inversely related, and KLF4 mRNA expression is significantly associated with androgen receptor (AR) expression [33]. These results suggest that low DYRK2 expression induces KLF4 and CSC formation via AR expression [33].

DYRK2 expression is inversely related to TERT [34] and cyclin-dependent kinase 14 (CDK14) expression in the breast cancer tissues of invasive ductal carcinoma [35].

High nuclear levels of DYRK2 and heat shock factor 1 (HSF1) have been detected in triple-negative breast cancer (TNBC) tissues, and high nuclear DYRK2 levels significantly reduced cancer-specific survival in TNBC and triple- and AR-negative patient samples [36].

Studies using cell lines have facilitated the discovery of DYRK2 functions. Low DYRK2 expression upregulates CD44^+^/CD24^−^ and ALDH1^+^ CSCs in pulmonary metastases of breast cancer [33]. A stem cell phenotype was detected by analyzing the CD44^+^/CD24^−^ subpopulation. Mammosphere assays revealed that DYRK2 knockdown accelerated mammosphere formation and increased the CD44^+^/CD24^−^ subpopulation [33]. Therefore, DYRK2 expression is inversely related to the number of CSCs in breast cancer specimens and cell lines [33].

KLF4 expression was significantly increased in mammospheres and was high in DYRK2 knockdown cells [33]. In addition, the double knockdown of DYRK2 and KLF4 reduced the subpopulation of CD44^+^/CD24^−^ and significantly reduced mammosphere formation [33]. KLF4 knockdown cells also exhibited decreased tumor formation [33]. Moreover, AR depletion decreased KLF4 expression in DYRK2 knockdown cells, and AR inhibitor treatment decreased mammosphere formation and KLF4 expression. Therefore, AR-regulated KLF4 transcription is dependent on DYRK2 [33].

DYRK2 downregulates TERT expression, and ectopic DYRK2 expression suppresses TERT expression in a dose-dependent manner [37]. A glutathione S-transferase pull-down assay revealed that the TERT protein binds to DYRK2 directly, and a kinase assay confirmed that DYRK2 phosphorylation at TERT-Ser457 induces TERT degradation [37]. The TERT–DYRK2 interaction was mainly detected in G2/M phase-arrested cells, and decreased interaction was detected in S-phase-arrested cells [37]. DYRK2 is involved in ubiquitination; DYRK2 functions as a scaffolding protein for the E3 Ligase complex EDD, DDB1, and VprBP (EDVP), which are ubiquitination substrates [38].

According to the COSMIC database, DYRK2 mutations have been identified in human breast cancer through the genome-wide analysis of various cancers [37,39,40,41]. DYRK2-S471X nonsense mutations failed to downregulate TERT or induce constitutive telomerase hyperactivation [37].

DYRK2 overexpression in 293T cells increases endogenous HSF1 phosphorylation at Ser320 and Ser326, which induces HSF1 activation [36]. DYRK2 overexpression increases nuclear HSF1 accumulation, which is decreased by DYRK2 knockdown [36]. Moreover, in HSF1 mutants, both Ser320 and Ser326 were mutated to alanine and were not phosphorylated upon DYRK2 overexpression [36]. DYRK2 knockout TNBC cells showed reduced HSP 70, a target gene downstream of HSF1, which was rescued by DYRK2 transfection [36]. DYRK2 knockdown in TNBC cells suppressed HSF1 nuclear stability; therefore, DYRK2 may contribute to HSF1 nuclear stability [36]. The response of DYRK2 knockout TNBC cells to heat shock is characterized by decreased cell viability and increased levels of cleaved PARP, a marker of apoptosis [36].

Microarray analysis revealed significantly higher CDK14 expression in DYRK2 knockdown MCF-7 cells than in control cells [35]. DYRK2 knockdown MCF-7 cells show enhanced cell proliferation, whereas stable DYRK2 overexpressed MDA-MB-231 cells show suppressed CDK14 expression and proliferation [35]. Both DYRK2 and CDK14 knockdown cells showed significantly decreased cell proliferation and invasion; these results were also observed in a xenograft mouse model [35]. Therefore, DYRK2 regulates cell proliferation and invasion via CDK14 [35].

DYRK2 phosphorylates Snail and regulates EMT, cell invasion, and metastasis via Snail expression [42]. DYRK2 expression promotes mTOR degradation and ubiquitination via Thr631 phosphorylation [43].

**Figure 5:**
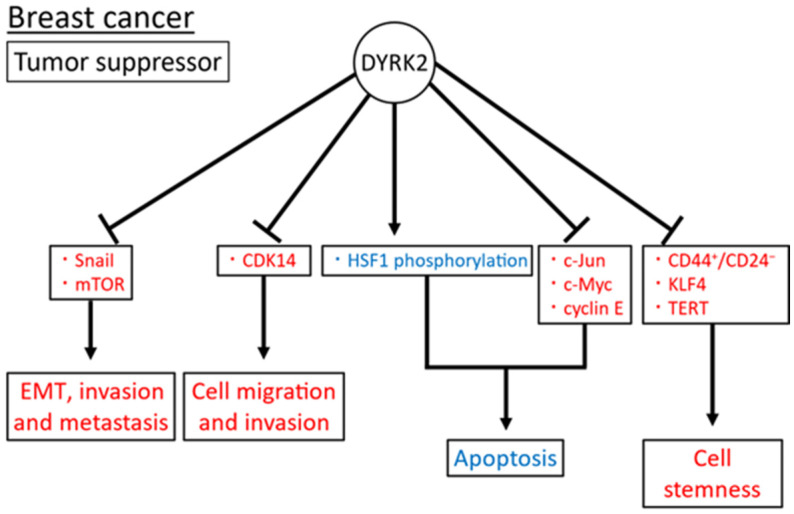
Reported DYRK2 (a tumor suppressor) substrates and functions in breast cancer. Induced and inhibited substrates or functions of DYRK2 are in blue and red, respectively. The arrow means promotion and the bar means inhibition respectively.

Taken together, DYRK2, as a tumor suppressor, may induce apoptosis by c-Jun, c-Myc, and HSF1 phosphorylation and inhibit cell stemness by suppressing the CD44^+^/CD24^−^ subpopulation and KLF4 and TERT expression via AR; cell proliferation via the suppression of CDK14 expression; and the regulation of EMT, invasion, and metastasis through the Snail phosphorylation and induction of mTOR degradation (Figure 5). Notably, the role of DYRK2 in breast cancer has been investigated in tissue samples, cell lines, and xenograft mouse models. However, to date, no such studies have been conducted using genetically modified mice.

These reports indicate that DYRK2 is a tumor suppressor gene in breast cancer. However, the following reports demonstrate the opposing role of DYRK2 as an oncogene.

Based on single-cell RNA sequencing datasets, a clear overlap between DYRK2 and HSF1 was detected in the clustered cell populations of renal cell carcinoma, colon adenocarcinoma, breast cancer, and astrocytoma [44]. In addition, the overlapping expression of DYRK2 and HSF1 was observed across all cancers in TCGA database [44]. High protein expression levels of DYRK2 in the nucleus result in local and distal recurrence in patients with TNBC [44]. Moreover, distal recurrence in patients with quadruple-negative breast cancer was shorter than that in patients with low protein expression levels of DYRK2 [44].

DYRK2 knockdown significantly reduced Rpt3-T25 phosphorylation (Thr25 of the 19S subunit) [45]. In addition, cells deficient in Rpt3-T25 phosphorylation showed reduced proliferation and proteasomal activity [45]. Rpt3-T25 is evolutionarily conserved among vertebrates and the DYRK2 consensus motif is conserved near the Rpt3-T25 sequence [45]. Strong Rpt3-25T phosphorylation was detected in the 26S proteasome treated with DYRK2, and increased proteasome activity was observed compared with that in the inactive DYRK2 mutant [45]. In contrast, DYRK2 did not stimulate the mutant 26S proteasomes purified from Rpt3-T25A knock-in cells [45]. Therefore, the 26S proteasome may be directly activated by DYRK2 [45].

DYRK2 knockout in MDA-MB-468 cells suppressed cell proliferation and delayed the cell cycle [45]. In contrast, DYRK2 overexpression downregulated the cell cycle inhibitors p21^Cip1^ and p27^Kip1^ [45]. These results suggest that DYRK2 positively regulates cancer cell growth [45]. It has also been reported that curcumin, an active ingredient in *Curcuma longa*, may be a DYRK2 inhibitor and decreases 26S proteasome activity through DYRK2 inhibition in TNBC and multiple myeloma cell lines, thereby inhibiting tumor proliferation [46]. In addition, DYRK2 knockout MDA-MB-231 cells showed a reduced tumor burden in xenograft mouse models, which was recovered through DYRK2 reintroduction [47].

**Figure 6:**
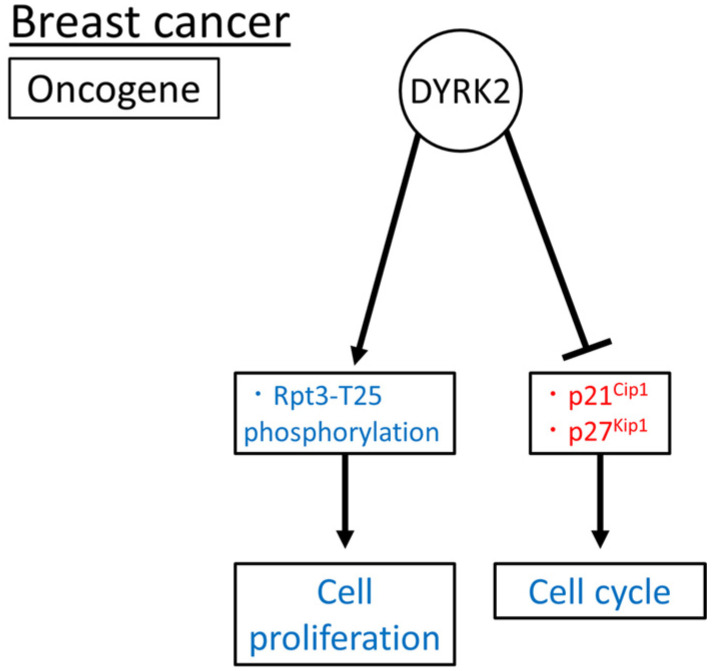
Reported DYRK2 (an oncogene) substrates and functions in breast cancer. Induced and inhibited substrates or functions of DYRK2 are in blue and red, respectively. The arrow means promotion and the bar means inhibition respectively.

Taken together, DYRK2, an oncogene, may induce cell proliferation via Rpt3-T25 phosphorylation and promote cell cycle progression by downregulating p21^Cip1^ and p27^Kip1^ (Figure 6). The role of DYRK2 in breast cancer has been investigated in tissue samples, cell lines, and xenograft mouse models. However, to date, no such studies have been conducted using genetically modified mice.

### 2.7. Lung Cancer

DYRK2 expression significantly decreased in tissue samples from patients with lung cancer [48]. In contrast, another study reported no relationship between DYRK2 expression and any clinical factors using immunohistochemistry; however, strong DYRK2 expression was detected in the cytoplasm of lung adenocarcinoma (LADCs) cells [49]. DYRK2 expression is higher in node-negative lymphatic invasion than in node-positive lymphatic invasion [49]. The five-year disease-free survival and overall survival of patients with DYRK2 positive expression were better than those with DYRK2 negative expression [49].

Taken together, DYRK2, as a tumor suppressor, may inhibit tumor invasion and contribute to better five-year disease-free and overall survival rates. Notably, the role of DYRK2 in lung cancer has been investigated only in patient tissue samples. Cell lines, xenograft mouse models, or genetically modified mouse models have not been used.

Other studies have reported opposing functions of DYRK2. Strong DYRK2 expression was detected via micropapillary (mPAP) staining in three *EGFR*-mutated LADCs from patient tissue samples [50]. *EGFR*-mutated or non-*EGFR*-mutated LADCs with mPAP showed a stronger relationship with DYRK2 expression than those without mPAP; however, the difference was not statistically significant [50]. In addition, DYRK2 has a high DNA copy number and mRNA overexpression in lung tumors [9]. The over two-fold increased upregulation of DYRK2 mRNA was found in 18.6% of 86 LADCs using an oligonucleotide microarray [9]. DYRK2 mRNA overexpression has also been reported to occur more frequently than gene amplification [9].

Together, these data revealed higher DYRK2 expression in tumors. However, cell lines, xenograft mouse models, and genetically modified mouse models have not been used.

### 2.8. Ovarian Cancer

Studies have shown an inverse relationship between DYRK2 and Snail expression [51]. In addition, patients with low DYRK2 expression in ovarian serous carcinoma have shorter overall survival [51].

It was reported that DYRK2 phosphorylated the Ser104 of Snail, which primes the GSK3 β-βTrCP-mediated Snail ubiquitination to inhibit EMT [52]. Therefore, DYRK2 may regulate EMT via Snail expression.

Taken together, DYRK2, as a tumor suppressor, may inhibit metastasis via Snail phosphorylation and contribute to improved overall survival. The role of DYRK2 in ovarian cancer has been investigated in tissue samples, cell lines, and xenograft mouse models. However, to date, no such studies have been conducted using genetically modified mice.

Another study reported that EDD, DYRK2, and E-cadherin expression was higher in tissue samples from patients resistant to the conventional chemotherapeutic agent cisplatin than in cisplatin-responsive samples [53]. In contrast, the expression of modulator of apoptosis protein 1 (MOAP-1) was suppressed in cisplatin-resistant samples, but the difference was not significant. This study investigated cell lines and reported that the EDD function of the EDVP E3 ligase complex (DYRK2 acts as a scaffold protein for this complex) may regulate MOAP-1 ubiquitination [53].

These studies revealed higher DYRK2 expression in cisplatin-resistant tumors. The role of DYRK2 as an oncogene in ovarian cancer was investigated using patient tissue samples and cell lines. However, to date, no xenograft or genetically modified mouse models have been developed.

### 2.9. Prostate Cancer

One study reported that prostate cancer tissue samples have markedly lower DYRK2 expression levels than normal tissue samples [18].

Low DYRK2 expression was observed in these tumors. Cell lines, xenograft mouse models, or genetically modified mouse models were not used.

Another study reported high DYRK2 expression in tissue samples from patients with prostate cancer. Furthermore, patients with low DYRK2 expression exhibited better relapse-free survival than those with high DYRK2 expression [54].

DYRK2 knockdown cell lines showed significantly reduced cell proliferation, migration, and invasion and induced G0/G1 cell cycle arrest [54]. In addition, tumor growth was inhibited by DYRK2 downregulation in xenograft mouse models [54].

Taken together, DYRK2, as an oncogene, may induce cell proliferation, migration, and invasion; promote cell cycle progression; and improve relapse-free survival. The role of DYRK2 in prostate cancer has been investigated in tissue samples, cell lines, and xenograft mouse models. However, to date, no such studies have been conducted using genetically modified mice.

## 3. Conclusions and Future Prospects

DYRK2 has been reported to regulate apoptosis and DNA repair induction, metastasis inhibition, cell cycle G1/S transition, protein stability as a scaffold protein for EDVP E3 ligase complexes, and embryogenesis [2,3,4,5,6,7,8,55]. Therefore, DYRK2 may act as a tumor suppressor. Furthermore, no cancer studies have reported that DYRK2 functions only as an oncogene, with the exception of esophageal cancer. However, the roles of DYRK2 as both a tumor suppressor and oncogene have been reported in some cancers. Overall, it is difficult to determine whether a sufficient number of studies have been published to determine the function of DYRK2, particularly as an oncogene in cancer. Further studies are required to validate the function of DYRK2.

Tissues from patients with different cancers were studied to evaluate the role of DYRK2 as a tumor suppressor and oncogene. Cell line and/or xenograft mouse model studies have been conducted to elucidate the mechanism of DYRK2 function in certain cancers. Although substrates of DYRK2 are important for understanding the mechanism underlying DYRK2 function, they remain unclear. In addition, only one study reported results from genetically modified mice [24] (Table 1). Studies using genetically modified mice are necessary to understand the precise function of DYRK2.

Genetically modified mouse models have provided information regarding the function of DYRK2 in various cancers. Cell line and patient tissue studies have provided much information about DYRK2 function; however, these experiments were conducted in vitro and need to be validated in vivo. Furthermore, cancer cell lines and patient tumor tissues are cancerous; therefore, it is difficult to determine whether tumorigenesis and/or tumor development cause changes in DYRK2 expression or whether changes in DYRK2 expression cause tumorigenesis and/or tumor development. Relationships between DYRK2 and other genes have been reported in vitro. However, it remains unclear whether the symptoms of related gene changes are detectable before tumorigenesis in vivo. Moreover, the development of a new cancer drug that targets DYRK2 requires animal experiments to determine drug efficacy, the presence or absence of side effects, and delivery routes. Therefore, animal experiments are necessary to elucidate the functions of DYRK2.

One of the main purposes of this review on DYRK2 was to discuss the potential of DYRK2 as a new target for cancer treatment. Therefore, we performed a detailed investigation into the mechanism by which DYRK2 functions in different cancers both in vitro and in vivo to determine the exact role of DYRK2 in cancer.

## Figures and Tables

**Table 1:** Reported studies of each cancer.

	The Role of DYRK2	Patients’ Tissues	Cell Lines	Xenograft Mouse Model	Genetically Modified Mouse
Colorectal cancer	Tumor suppressor	✓	✓	✓	-
Liver cancer	Tumor suppressor	✓	✓	✓	✓
Gastric cancer	Tumor suppressor	✓	✓	✓	-
Non-solid tumor (leukemia)	Tumor suppressor	-	✓	-	-
Cancer stem cells	Tumor suppressor	-	✓	✓	-
Breast cancer	Tumor suppressor	✓	✓	✓	-
	Oncogene	✓	✓	✓	-
Lung cancer	Tumor suppressor	✓	-	-	-
	Oncogene	✓	-	-	-
Ovarian cancer	Tumor suppressor	✓	✓	✓	-
	Oncogene	✓	✓	-	-
Prostate cancer	Tumor suppressor	✓	-	-	-
	Oncogene	✓	✓	✓	-

Check marks indicate the presence of a report and hyphens indicate its absence.
